# Potential Therapeutic Role of Mesenchymal-Derived Stem Cells as an Alternative Therapy to Combat COVID-19 through Cytokines Storm

**DOI:** 10.3390/cells11172686

**Published:** 2022-08-29

**Authors:** Tarun Kumar Upadhyay, Rashmi Trivedi, Fahad Khan, Pratibha Pandey, Amit Baran Sharangi, Harsh Goel, Mohd Saeed, Moon Nyeo Park, Bonglee Kim

**Affiliations:** 1 Department of Biotechnology, Parul Institute of Applied Sciences and Animal Cell Culture and Immunobiochemistry Lab, Centre of Research for Development, Parul University, Vadodara 391760, India; 2 Department of Biotechnology, Noida Institute of Engineering & Technology, Greater Noida 201306, India; 3 Department of Plantation, Spices, Medicinal & Aromatic Crops, BCKV-Agricultural University, Mohanpur 741252, India; 4 Department of Laboratory Oncology, All India Institute of Medical Sciences, New Delhi 110023, India; 5 Department of Biology, College of Sciences, University of Hail, Hail 34464, Saudi Arabia; 6 Department of Korean Medicine, Kyung Hee University, Seoul 05254, Korea; 7 Department of Pathology, College of Korean Medicine, Kyung Hee University, Seoul 02447, Korea

**Keywords:** COVID-19, stem cell therapy, cytokine storm, mesenchymal stem cells, inflammation

## Abstract

Medical health systems continue to be challenged due to newly emerging COVID-19, and there is an urgent need for alternative approaches for treatment. An increasing number of clinical observations indicate cytokine storms to be associated with COVID-19 severity and also to be a significant cause of death among COVID-19 patients. Cytokine storm involves the extensive proliferative and hyperactive activity of T and macrophage cells and the overproduction of pro-inflammatory cytokines. Stem cells are the type of cell having self-renewal properties and giving rise to differentiated cells. Currently, stem cell therapy is an exciting and promising therapeutic approach that can treat several diseases that were considered incurable in the past. It may be possible to develop novel methods to treat various diseases by identifying stem cells’ growth and differentiation factors. Treatment with mesenchymal stem cells (MSCs) in medicine is anticipated to be highly effective. The present review article is organized to put forward the positive arguments and implications in support of mesenchymal stem cell therapy as an alternative therapy to cytokine storms, to combat COVID-19. Using the immunomodulatory potential of the MSCs, it is possible to fight against COVID-19 and counterbalance the cytokine storm.

## 1. Introduction

COVID-19, caused by the SARS-CoV2 virus, is a perilous disease that threatens global public health. “COVID-19”stands for Coronavirus Disease of 2019 and was named by the WHO on 11 February 2020 [1]. SARS-CoV-2, the causative agent of this disease, is an enveloped, positive, single-stranded RNA virus belonging to the beta-coronaviruses subfamily.

The spread of coronavirus occurs mainly through the droplets generated through the sneezing and coughing of the infected person [2]. Incubation period for this virus after transmission is from 2 to 14 days. The SARS-CoV-2 virus resides in the lower respiratory tract and causes pneumonia in humans, eventually leading to fatality from chronic hyper-inflammation and respiratory distress [3]. It attaches with the help of spike proteins present on its membrane, and mRNA coding for this spike protein induces mutations, making it antigenically favorable. The lipid nanoparticles protect the non-replicating RNA from degradation and allow it to be delivered into host cells. Once inside the host cell, the mRNA is translated into the SARS-CoV-2 spike protein, which is generated on the cell’s surface [4]. 

SARS-CoV-2 is a beta coronavirus belongs to the family coronaviridae and is mainly responsible for COVID-19. However, some of their mutants that arise mostly due to mutations are also responsible for causing COVID-19. SARS-CoV-2 family of coronavirus is closely related to the variant of coronavirus found in the population of bats and SARS-CoV. Two more variants named RaTG13 and RmYN02 show approximately 96.2% and 93.3% sequence homology, respectively, with the SARS-CoV-2 virus family and these variants are also found in the bat population [5,6]. Some of the variants such as B.1.526, B.1.525, and P.2 arise due to a common mutation D614G in which aspartic acid is replaced by glycine at codon 614, changing its spike protein. These variants have properties to spread faster than many other variants and have many different properties than original SARS-CoV-2 [7]. Despite this, some more variants such as 501Y.V1 (B.1.1.7) and 501Y.V2 (B.1.351) emerged in UK and South Africa, respectively. These variants had a mutation at the receptor-binding domain of the spike protein that helped in the higher spread of this variant. The 501Y.V2 variant arose due to additional mutations E484K and K417N of the spike protein in the 501Y.V [8,9,10]. In Southern California, a new variant named CAL.20C was reported to derive from 20C cluster and had a mutation in ORF1a: I4205V, ORF1b: D1183Y, spike protein: S13I, W152C, and L452R [11].

In COVID-19 disease, acute respiratory distress syndrome (ARDS) is the leading cause of death. Its main feature is the cytokine storm, an uncontrolled inflammatory response triggered by immune cells releasing cytokines and chemokines [12,13]. After infection with COVID-19, lung epithelial and endothelial cell apoptosis and vascular leakage, alveolar edema, and hypoxia result from the abnormal release of pro-inflammatory factors. ARDS is caused by an uncontrolled release of pro-inflammatory factors, such as IL-6, IL-8, and IL-1. In most severe patients of COVID-19, improper function of the immune system results in enhanced production of cytokines (cytokine storm) such as IL-2, IL-6, colony-stimulating factor, and TNFs, ultimately leading to death [13]. Furthermore, it is also caused by the release of chemokines and reactive oxygen species, such as CCL-2, CCL-5, IP-10, and CCL-3 [14]. Entry of the coronavirus in host cells occurs from receptor-mediated endocytosis through the numb-associated kinases (NKA). The capability of coronavirus to cause the disease resides in the spike glycoproteins, which binds to the angiotensin-converting enzyme-2 (ACE-2) receptor present on the alveolar epithelial cells, endothelial cells, and cardiac and renal cells. After binding with the receptor, the virus enters the cell’s cytoplasm to release its genetic material. Genetic material (RNA) replicates and gives rise to new virus progeny, resulting in the spread of the virus to the other cells due to cell burst [13,15].

Even though most COVID-19 patients are asymptomatic, some develop pneumonia, and about 10% require ventilation. As the most common symptoms, patients can have fever, cough, breathing difficulty, headaches, muscle and bone pain, hemoptysis, diarrhea, and nausea [3]. In 10–20% of the total infection cases of SARS-CoV2 virus, it may cause interstitial pneumonia and acute respiratory distress syndrome (ARDS), especially in older age people [16]. For the entry in lungs after infection, SARS-CoV-2 virus recognizes angiotensin I converting enzyme 2 receptor with the help of its spike proteins. After recognition, its spike protein primed by cellular transmembrane protease, serine 2 (TMPRSS2) leads to its entry and further spread to other organs [17,18,19]. SARS-CoV2 virus does not remain confined to the respiratory tract but can invade the CNS and induce many neurological diseases, causing severe illness. Moreover, SARS-CoV2 can also invade many organs simultaneously, resulting in multi-organ failure. Mesenchymal stem cells (MSCs) are emerging as therapy against SARS-CoV2 due to their distinctive ability to improve immune functions to combat multiple and severe disease conditions. MSCs show immunomodulatory effects by the secretion of a variety of paracrine factors. These paracrine factors interact with immune cells that lead to the immunomodulation [20]. In a study, it was reported that the infusion of umbilical cord-derived stem cells into patients having ARDS and cytokine storm resulted in better functional outcomes [21]. In another study, MSCs were implanted in a patient with severe brain and multiple organ infection along with developing cardiac arrest by COVID-19. It was reported that MSCs incorporation had a healing effect on infected organs and severe infection [22].

## 2. Cytokines

Small polypeptides of glycoproteins known as cytokines elicit diverse responses in the body by interacting with their receptors via autocrine, paracrine, or endocrine signaling. Cytokines can stimulate cellular proliferation, differentiate cell communication, and regulate immune responses based on the target cell type. The receptors bind to cytokines and subsequently alter gene transcription by triggering intracellular signaling. Cytokines can be growth factors, chemokines, or interleukins formed by superfamilies having familiar and different gene structures. They are pleiotropic, and different cytokines can have the same effects [23,24]. One of the largest classes of cytokines is chemokines accounting for nearly 44 members that play various roles in regulating the immune system, such as recruitment and trafficking of leukocytes. Any dysregulation in the trafficking mechanism can lead to hyperinflammation [25]. Table 1 shows various cytokines and their secretary cells along with their mode of action.

## 3. COVID-19 and the Cytokine Storm

Hyperactive host immune responses result in an excessive inflammatory response to the SARS-CoV-2 virus, popularly known as the “cytokine storm”. As far as we know, there is no universally accepted definition for cytokine storms or cytokine release syndromes. A study found that an auto-amplifying cascade of cytokines triggered by an immune system unregulated by different triggers such as infection, malignancy, and arthritis, can be a “cytokine storm” [76]. Similarly, another study suggested that cytokine storms are caused by systemic inflammation caused by infections and drugs and often result in excessive activation of the immune system and the release of pro-inflammatory cytokines [77]. A cytokine storm occurs when cytokines are released to be harmful to host cells. Dysregulated cytokine production damages healthy cells of the lungs, further spreading to the heart, kidney, vessels, and other organs. Moreover, cytokine storm may also depend on entry and binding of SARS-CoV-2 spike protein with membrane serine proteases of the host [78]. Entry of SARS-CoV-2 into respiratory epithelial cells induces immune cells along with the production of inflammatory cytokines due to the weak response of interferon (IFN).Downregulation of some immune system-associated signaling pathways regulate the immune response of pathogenic Th1 cells and CD14^+^CD16^+^monocytes that results in infiltration of macrophages and neutrophils in lung tissues, leading to the cytokine storm [79].

Cytokine storms can be readily identified in disorders with elevated cytokine levels. A complex question is whether certain cytokines help control infections while at the same time harming the host. This is mostly due to the fact that some cytokines help control infections while being harmful at the same time [80]. Cytokine storm is a broad term including characterization of immune system dysfunction by symptoms of inflammation leading to multi-organs failure in the case of inadequate treatment. Cytokine concentration may vary according to the cause and treatments against it. C-reactive proteins (CRPs) are considered as the diagnostic marker for inflammation. These CRPs are non-specific, and their elevated level indicates the severity of the disease [81,82]. In patients with severe COVID-19, C-reactive protein (CRP) levels in the blood are markedly elevated [83]. CRP is synthesized and released by the liver in response to the stimulation of interleukin-6. Researchers have identified the presence of both pro-and anti-inflammatory CRP, which can be used for monitoring the extent of tissue damage associated with the pathogenesis of COVID-19 [84].In a study by McElvaney et al., it was found that severe COVID-19 patients show higher levels of IL-1β, IL-6, and sTNFR1, but lower levels of IL-10 than the patient with mildly infected COVID-19 patients [85]. Levels of IL-6 and TNF-α in the serum can be considered for the COVID-19 patient treatment and management for the clinical trials, to guide resource allocation and therapeutic options [86].

Various immune cells such as macrophages and mast cells are responsible for the secretion of pro-inflammatory cytokines IL-1, TNF-α, IL-6, GSCF, IL-7, MIP1A, IL-2, and IP10, and chemokines such as CCL-2, CCL-3, CCL-5, CXCL-8, CXCL-9, and CXCL-10, leading to the innate immune response in the body [87,88]. The more-than-usual secretion of these pro-inflammatory cytokines attracts T cells, neutrophils, and macrophages, including many more immune cells to the site of infection from the circulation. These immune cells, in bulk, destabilize endothelial cell-to-cell interaction, cause damage to the capillaries and alveoli, and damage the vascular barriers, resulting in severe lung injury [89]. Cytokine storms develop symptoms according to the increased cytokines. Unregulated secretion of TNF-α and IFN-γ may result in fever, fatigue, vascular leakage, and lung injury. Another essential cytokine, IL-6, can cause coagulation leakage, complement system activation, and cause vascular leakage [90,91,92]. Adding to the complexity, most mediators involved in cytokine storms manifest pleiotropic downstream effects, and their biological activities are often interdependent. There will neither be a linear nor a constant interaction of these mediators. However, measurements of their quantitative levels are not always indicative of pathogenicity. Understanding this complex interplay allow us to know the limitations of targeting single mediators and intervening in the acute inflammatory response [93].

Interferons (IFNs) are the main secretory immune response to provide the defense against viral infection. IFNs work as the first line of defense against viral infection and help in viral clearance through the modulation of innate and adoptive immune systems. In the case of SARS-CoV-2 infection, an elevated level of IFNs can regulate the cytokine storms by removing the SARS-CoV-2 virus. IFNs regulate various signaling pathways such as nuclear factor-κB (NF-κB), IFN regulatory factor 3/7 (IRF3/7), and activator protein-1 (AP-1). Activation of these pathways further activates Janus kinase 1 (JAK1)/tyrosine kinase 2–signal transducer and activator of transcription 1/2 (STAT1/2) pathway. These activated pathways promote the formation of STAT1/2/IRF9 complex, resulting in increased production of IFN-stimulated genes (ISGs) [94,95].

## 4. Stem Cells and Stem Cell Therapy

The stem cells are believed to be precursors of diverse tissues capable of self-renewal and provide replacement cells for a broad range of tissue types. The inner cell mass of the embryonal blastocyst is the primary source of embryonic stem cells. Stem cells can also be isolated from different sources such as the umbilical cord, fetal liver, adipose tissues, and bone marrow. Many cytokines are synthesized and secreted by stem cells that stimulate cell recruitment, angiogenesis, immunomodulation, neuroregeneration, and extracellular matrix remodeling. In addition to generating various cell types, stem cells can differentiate into other types of cells, such as endothelial cells, pericytes, myofibroblasts, and keratinocytes, which may play a role in wound healing [96,97,98,99]. Stem cells can essentially be of three types: embryonic stem cells (ESCs), adult stem cells, and mesenchymal stem cells (MSCs). Embryonic stem cells can be isolated from the inner cell mass of the early embryo, and these cells have high regenerative potential. Adult stem cells can be isolated from various sources such as cord blood, placenta, bone marrow, peripheral blood, adipose tissue, and menstrual blood. One of the types of adult stem cells, MSCs, is very efficient types of stem cells, having therapeutic, immunomodulatory, and regenerative properties [100].

**Figure 1:**
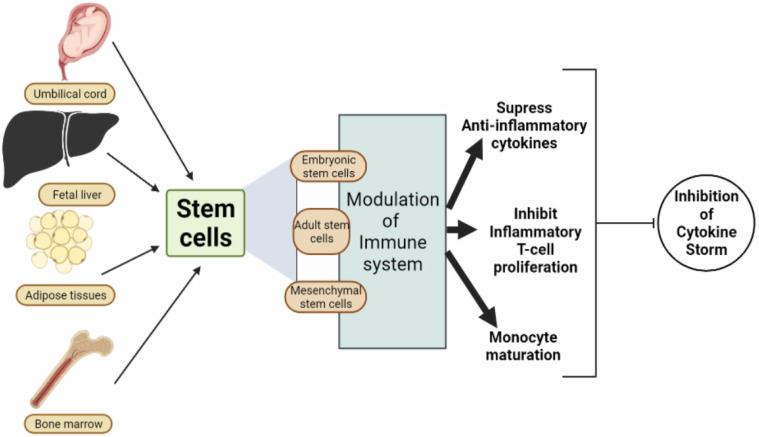
Modulation of immune system by different types of stems cells to prevent cytokine storm.

Compared to other therapeutic strategies, MSCs are viewed as more attractive since they are multipotent, have a high proliferation rate, and are free from social or ethical issues [101]. MSCs have a similar morphology to fibroblasts. Hence, it is difficult to identify them morphologically. There are, however, cellular markers that can help identify MSCs [102]. Because of their immune-evasive nature, MSCs release factors that allow them to remain immune from rejection mechanisms for an extended period, allowing them to have the desired therapeutic effect [103]. MSCs can give rise to several cells, such as stromal cells, myoblasts, adipocytes, osteoblasts, endothelial cells, and chondrocytes [104]. MSCs exhibit anti-inflammatory and immunomodulatory benefits by expressing anti-inflammatory cytokines, inhibiting inflammatory T-cell proliferation, and inhibiting monocyte maturation as shown in Figure 1 [105,106,107,108]. MSCs are plastic-adherent stromal cells expressing biomarkers such as CD1025, CD73, and CD90, and devoid of a few biomarkers, including CD45, CD11b, CD19, and many more [109]. The properties of human mesenchymal stem cells, such as the production of paracrine factors VEGF (vascular endothelial growth factor), FGF (fibroblast growth factor), and HGF (hepatocyte growth factor), which promotes angiogenesis, neovascularization, and cell survival, have made them a well-known candidate for cell-based therapies for many years [110]. Due to the potential use of mesenchymal stem cells (MSC) in autologous transplantation, these cells are of great clinical interest. MSCs have been used in several trials, including this one, and many others are undergoing testing. Recently, reports revealed that 2000 patients were treated with allogeneic or autologous MSCs for various diseases by autologous or culture-expanded MSCs [111].

In recent years, studies have emphasized the paracrine properties of MSC and the mechanism of release of extracellular vesicles containing mRNAs, regulatory molecules, bioactive molecules, and the production of regulatory substances overall, rather than on the direct differentiation and replacement of cells by MSC [112,113]. A new cellular therapy that uses mesenchymal stem cells from bone marrow is BM-MSCs. However, clinical implementation of these BM-MSCs still remains challenging. Although the first generation infusion of BM-MSCs was found to be safe according to meta-analysis, still many uncertainties exist, such as hemato-compatibility, side effects of large doses, and safety of adipose tissue and perinatal tissue-derived products [114,115,116,117,118,119]. Mesenchymal stem cells can be beneficial for generating many kinds of organs and treating various diseases. Genomic alterations in these MSCs can improve survival rate, growth factor secretion, and increased migration [120]. MSCs can modulate the immune system, leading to the varied responses of immune cells. They can inhibit T-cells’ cytotoxicity and proliferation, resulting in the inactivation of T-cells [121]. Table 2 shows the stem cells used as a therapy for various diseases and their mode of action.

## 5. Stem Cell Therapy for COVID-19 and Cytokine Storm

**Figure 2:**
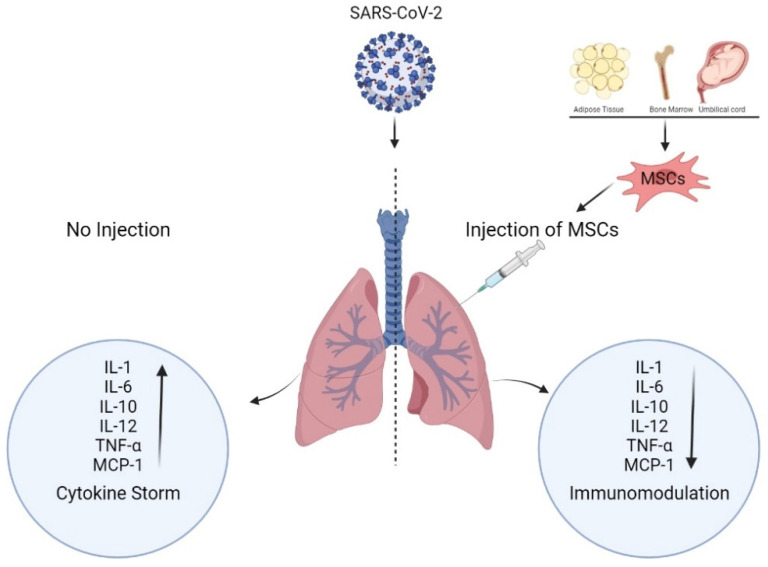
Effects of mesenchymal stem cells (MSCs) therapy on the patients of COVID-19.

As infection of COVID-19 is increasing globally with its new variants Omicron and XE, this is high time to find a complete treatment apart from the vaccine to prevent COVID-19. The XE variant is a combination of BA.1 and BA.2 variant of omicron in which BA.2 is already spreading 10% faster thanBA.1 variant [144]. Many studies are going on to explore the role of stem cells in suppressing the cytokine storm during COVID-19, as MSCs are found to have an efficient immunomodulatory role [100]. Various studies related to COVID-19 and cytokine storm have demonstrated that cytokine levels vary in COVID patients according to the severity of the disease. Patients with less coronavirus load expressed low levels of inflammatory cytokines and enhanced levels of epidermal growth factor (EGF), platelet-derived growth factor (PDGF), and vascular endothelial growth factor (VGEF); while patients with a heavy dose of coronavirus expressed a higher level of pro-inflammatory cytokines [145]. MSCs therapy in the case of stem cell therapies is an efficient therapy to treat various diseases, and COVID-19 is one of them. MSCs express various surface markers such as CD-73, CD-90, and CD-105, having the ability to differentiate into MSCs progeny, which are minimal criteria for defining multipotent mesenchymal stromal cells [109]. MSCs have many cell surface markers such as CD146 and CD200, which are unique and non-differentiating in nature. They also possess some matrix and MSC markers such as CD29, CD44, CD71, CD73, and CD105. These markers give MSCs immunotolerant and immunomodulant properties in damaged tissues along with regenerating and rejuvenating properties by exerting their effects on immune cells including T and B lymphocytes, dendritic cells, and macrophages [146,147,148]. SARS-inflammatory Cov-2 response can be used as a primary approach for eliminating the virus. SARS CoV-2 entry results in the release of pro-inflammatory molecules such as interleukin (IL), tumor necrotic factors alpha (TNF-α), and multiple interferons (IF). These can restore and control the immune system, which is helpful for cell therapy [149]. Different investigations have revealed that impairment of mesenchymal stem cells (MSCs) raises the entry of viruses and their pathogenicity [150]. In addition, MSCs mechanism modulates the immune system; these cells have the right to regulate the growth and function of immune cells by reducing the production of TNF-α; MCP1; and anti-inflammatory cytokines such as IL-10 and 12, which result in reduced differentiation and block dendritic cells by generating inflammation and activation of different immune cells [151,152]. As shown in Figure 2, a Mesenchymal stem cell (MSCs) injection in a patient suffering from COVID-19 reduces the secretion of interleukins and inflammatory factors, which prevent further SARS-CoV-2 infection and can be used as a viable alternative treatment option [153].

MSCs have a high proliferation rate and the least social and ethical issues. Due to their ability to self-renew and to differentiate into multiple cell types, stem cells are attractive as an option in cell therapy in the clinic. These cells can be easily obtained from various sources such as fatty tissues, umbilical cord, fetal liver, and bone marrow [101]. Although, stem cell therapies have made some progress, they still have remained relatively slow due to ethical and legal restrictions [154]. MSCs might activate the immune system to prevent the exaggerated release of cytokines, chemokines, and reactive immune cells, resulting in endogenous repair. In a study, researchers performed 10x RNA sequencing to understand the mechanism of MSCs action on COVID-19. In MSCs, ISGs (interferon-stimulated genes) played a significant role in their resistance to viral infections compared to their differentiated descendants [155]. ISGs prevent viral infection by expressing themselves. MSCs can inhibit the action of excessively active immune cells by releasing various cytokine and anti-inflammatory factors including TGF-β and prostaglandin E2 (PGE2) [156]. Moreover, MSCs can enhance the production of lymphocytes and regulatory dendritic cells to increase their antiviral characters that can ultimately lead to a decrease in pro-inflammatory cytokines such as IL-6, IL-8, and TNF-α. These cytokines are the main markers of inflammation and reactive oxygen species to decrease the oxidative stress and inflammation [157]. MSCs can also protect the alveolar epithelial cells by reducing inflammation and normalize the lung functions by modulating pulmonary microenvironment and inhibiting pulmonary fibrosis [158]. MSCs can stimulate alveolar stem cells to repair and regenerate healthy lung parenchyma cells [159]. In addition, many secreted factors having pharmacological effects from MSCs can be used to treat COVID-19. Genetic modification of MSCs to make them able to secrete bioactive molecules is another approach that can be used to treat COVID-19 [160]. Moreover, MSCs can inhibit the abnormally activated T-cells and macrophages, and apart from this, they also can turn them into regulator T-cells and anti-inflammatory macrophages. MSCs prevent pro-inflammatory cytokines from secreting as well, thereby reducing cytokine storms [161]. Apart from the immune system modulation, MSCs hinder the differentiation of monocytes into dendritic cells (DC), resulting in the downregulation of inflammatory cytokines and upregulation of regulatory cytokines. Leng and colleagues recently published an investigation of MSCs in COVID-19 patients from China. Specifically, seven out of ten patients were given intravenous infusions of clinical-grade allogeneic MSC, while the other three patients received saline as a placebo. Two of three patients developed ARDS in the placebo-treated group or expired after 14 days, but rest seven MSC-treated patients recovered. On comparing the MSC-treated group with the placebo group, there was a noticeable reduction in systemic inflammation with a 10-fold decrease in CRP levels, a lower TNF-α level, and increased IL-10 levels [17]. Furthermore, a decrease in serum levels of pro-inflammatory cytokine TNF-α and an increase in anti-inflammatory cytokine IL-10 in patients with COVID-19 following MSC transplantation suggests efficient regulation of cytokine storms [162].

## 6. Challenges in Stem Cell Therapy

The main challenge in stem cell therapy is the isolation and culture of MSCs. The donor’s age is an essential factor for transplantation because it becomes difficult to obtain an efficient number of MSCs from an aged donor. Apart from age, genetic traits and the donor’s medical history are also essential to consider. Moreover, if a donor obtains MSCs with any form of disease such as diabetes, a loss of function of these cells can be seen [163,164,165,166]. Although MSCs are the safest population of stem cells having negligible risk of endogenous teratogenic potential, some of the MSCs can lead to adverse effects after their in-vivo transplantation [167]. Moreover, MSCs’ immunomodulatory properties are generally not recommended for use in infectious diseases, especially in bacterial infections, which require a robust immune response to eliminate. It was surprising to find out that preclinical evidence indicated that MSCs could enhance antibacterial processes and decrease overactive immune responses, resulting in lethal acute respiratory distress syndrome (ARDS) [168].

## 7. Conclusions

COVID-19 is a globally emerging public threat, and treatment of the severely infected patient is an international issue of consideration. The prevention and treatment of COVID-19 are ongoing through many therapies, but a complete cure is yet to come. MSCs emerged as an attractive and readily available source that can be further processed to overcome COVID-19 and cytokine storm. However, there are some barriers such as donor heterogeneity, lack of in vitro expansion, and absence of standard procedures to manipulate cells, limiting the potential use of MSCs as therapy against COVID-19 and cytokine storm. Apart from some limitations, MSCs have plasticity and a huge immunomodulatory effect resulting in anti-cytokine storm therapy. If it becomes possible to standardize the therapeutic procedures and sources of MSCs, it will be possible to deal with the COVID-19 and cytokine storm with the help of stem cell therapy.

## Figures and Tables

**Table 1:** Cytokines, their secretary cells, and mode of action.

Family	Cytokine, Pro-Inflammatory Factor	Secreted by	Types of Cells on Which It Acts/Function	Mode of Action/Mechanism	References
Cytokine	GSCF (Granulocyte Colony-Stimulating Factor)	Endothelium, macrophages	Mouse lymphoid-biased	Anti-apoptotic, angiogenic, neurogenesis and functions.	[26,27,28,29]
Cytokine	IP10	Monocytes, T-cells, endothelial cells, and keratinocytes	It recruits immune cells to fight at inflammatory sites	To stimulate apoptosis, chemotaxis, cell growth, and angiostasis	[26,30,31,32]
Chemokines	MCP1 (Monocyte Chemoattractant Protein 1)	Microglial cells, mesangial, epithelial, smooth muscle, astrocytic, monocytic, and endothelial	Attracts T- lymphocytes, monocytes, and natural killer cells	It infiltrates, facilitates the migration of inflammatory cells and other cytokines towards the site of Inflammation.	[26,33,34,35]
Chemokines	MIP1A (Macrophage Inflammatory Protein 1 α)	Monocytes and macrophages	Act upon inflammatory cells and maintain impulsive immune response.	Healing wounded cells and halting stem cells.	[36,37]
Cytokine	IL-2	CD4+ T cells	Act against microbial infection as a natural impedance. It also promotes T cells differentiation into an effector T cell and then into memory T cell as the incident with antigen.	Ameliorate AICD (Activation Induced Cell Death) and increase the killing activity of T_c_ (Cytotoxic T) cells and NK cells.	[38,39,40]
Cytokine	IL-6	Dendritic cell and macrophages	Inflamed acute-phase protein synthesis, neutrophile in bone marrow, and help in the growth of B-cells.	IFN-γ secretion is affected by IL-6 through CD4 T cells, i.e., curial interferon that uplifts, IL-6 triggers CD4 cells to release IL-4 and directly affects Th2.	[41,42,43]
Cytokine	IL-7	Stromal cells in thymus and bone marrow	It affects mature T-cells and immature B-cells and leads to secondary cytokine release.	It involves mechanically on TCR-gamma and TCR-gamma delta thymocyte maturation.	[44,45]
Cytokine, superfamilyTNF	TNF-α (Tumor necrosis factor α)	Macrophages/monocytes	Perform miscellaneous functions within the cells during acute inflammation, and it activates and proliferates naïve and effector T cells.	Diverse signaling pathways lead to necrosis or apoptosis.	[46,47,48]
Chemokine (CXC Family)	IL-8	Mainly by macrophages /monocytes and some other cell types like epithelial cells, endothelial cells, smooth muscle cells, and airways	It has a direct effect on immune cells and polymorphonuclear cells.	IL-8 is considered a prognostic and therapeutic factor for wound healing.	[49,50,51]
Eicosanoid inflammatory mediators	Leukotriene (LT)	Mast cells	Create inflammatory cascade, effect on leukocytes, and stenosis of smooth muscles.	Their mode of action depends on the effective binding with G-protein-coupled receptors, and every LT receptor has an abnormal expression pattern and function.	[52,53,54,55]
Cytokine	IL-1β	Dendritic cell, activated macrophages	Pro-inflammatory cytokine and held in inflammation, autoimmune conditions, and pain.	IL-1β binds to the IL-1 type 1 receptor (IL-1R1), leads to the illustration of inflammation, and has the potency to induce fever when delivered exogenously.	[56,57,58]
Cytokine	IL-12	Dendritic cells	IL-12 receptors are present on T cells and NK cells, stimulating TH1 and NK cell growth while inhibiting TH2 cell responses.	This molecule produces interferon (IFN-γ), encourages the differentiation of T helper 1 (TH1) cells, and provides a link between innate defenses and adaptive defenses.	[59,60,61]
Cytokine	IL-33	Cellular damage area of bronchial epithelial cells, airway, endothelial cells of high endothelial venules	Generally, mast cells become degranulated when exposed to IL-33, and the effect also occurs in basophils and granulocytes.	It enhances Th2 responses.	[62,63,64,65]
(TGF-β) family	TGF-β	Monocytes/macrophages, lymphocytes and platelets	In addition to interacting with the surrounding cells, this TGF-β acts on smooth muscle cells, immune cells, and endothelial cells.	The condition causes angiogenesis and immunosuppression, which makes cancer more aggressive.	[66,67]
CC Family Chemokine Scavenger Receptor	CXCL-10	Dendritic cell and macrophages	This protein controls the differentiation of naive T cells into T helper 1 (Th1) cells and mediates immune cell migration to the foci.	This CXCL-10 chemokine binds to the CXCR-3 receptor to produce its effects in the cell.	[56,68,69,70]
Signaling proteins	IF	Natural killer (NK) cells, activated T cells, dendritic cells and macrophages.	Several cells, including monocytes, macrophages, T-lymphocytes, glia, and neurons, have IFN receptors.	When IFN-γ is produced, its effects are antiviral, antimicrobial, antitumor, and immunomodulatory. IFN proteins beta, alpha, and gamma are what produce those effects.	[56,71,72,73]
Cytokine	IL-18	Monocyte/macrophage	IL-18 activates th1 cells, and CD8+ T and natural killer (NK) cells are enhanced by it.	It increases the cytotoxic activity of CD8+ T cells and NK cells by upregulation of FasL.	[74,75]

**Table 2:** Stem cell type, source, and their mechanism of action.

S. No.	Stem Cell	Type of Cell	Isolated from Which Portion	Mode of Action	References
1.	Mesenchymal Stem Cell (MSC)	Multipotent stem cells	Fetal liver, bone marrow, umbilical cord, menstrual blood, dental pulp, adipose tissues, etc.	They perform an endogenous repair of stem cells and prevent the excessive release of cytokines from the immune system.	[122]
2.	Hematopoietic Stem Cells (HSCs)	HSCs are pluripotent and have ambient self-renewal efficiency.	HSCs are predominantly found in the bone marrow region, sternum, femur portion, umbilical cord, and even in a few segments of peripheral blood.	Regulated in two forms of mechanism. The first mechanism says they control the G0 phase, and in another mechanism it is fate determination, i.e., either differentiation or self-renew)	[123,124,125,126,127]
3.	Epithelial Stem Cells (ESCs)	ESCs are multipotent stem cells due to self-renewal capability throughout the life and/or unipotent progenitor cells.	They were isolated from the different layers of skin, i.e., from ectoderm, mesoderm, and endoderm.	In its action, various cellular-signaling mechanisms take parts, such as bone morphogenetic protein, WNT, and Sonic Hedgehog, which play a prominent part. These signaling pathways govern the conserved mechanisms behind the self-renewal capability of adult epithelial structures.	[128,129]
4.	Neural Stem Cells (NSCs)	They are self-renewal and multipotent stem cells,	In the adult mammalian brain, the sub-granular zone and subventricular zone have the reservoir of NSCs.	The formation of new hippocampal NSCs and its cellular mechanism taking part in it, along with a decrease in neurogenic potential is still unclear and therapeutic cargoes exchange in horizontal to host cell through extracellular vesicles is also not fully understood.	[130,131]
5.	Embryonic Stem Cells (ESCs)	The ESCs or human embryonic stem cells (hESC) possess tremendous pluripotent property and an extraordinary proliferative and growth capacity.	These ESCs are isolated from the mammalian blastocyst.	The ESCs mechanism of action depends on transcription factors associated with four genes viz., Sox2, Oct4, Tcf3, and Nanog that maintain pluripotency.	[132,133,134]
6.	Adult Stem Cells (ASCs)	These are multipotent, undifferentiated cells that renew themselves and preclude them into specialized cell types.	ASCs can be isolated from blood, bone marrow, skin, adipose tissue, and liver.	Due to environmental stimuli, ASCs release biologically active compounds that lead to exerting paracrine action on different neighboring cells and hence leading to repair, tissue protection, regeneration, self-renewal, and proliferation taking place.	[135,136,137,138]
7.	Induced Pluripotent Stem Cells (iPSCs)	These are (iPSCs) genetically engineered from somatic cells and pluripotent.	These are isolated from human adult somatic cells.	The remarkable feature of iPSCs to differentiate it into required specialized cell types and this property provides a source for innovative cell therapies with unlimited cell sources.	[139,140,141,142]
8.	Umbilical cord-derived MSCs	They are multipotent stem cells.	Isolated from the human embryo.	The mechanism of action (MOA) is still unknown	[143]

## Data Availability

Not applicable.

## Abbreviations

ARDS	Acute respiratory distress syndrome
MSCs	Mesenchymal stem cells
IL	Interleukin
(LT)	Leukotriene
CRPs	C-reactive proteins
ESCs	Embryonic stem cells
VEGF	Vascular endothelial growth factor
FGF	Fibroblast growth factor
TNFs	Tumor Necrosis Factors
HGF	Hepatocyte growth factor
HSCs	Hematopoietic Stem Cells
ESCs	Epithelial Stem Cells
NSCs	Neural Stem Cells
NKA	Numb-associated kinases
ACE-2	Angiotensin-converting enzyme-2
ASCs	Adult Stem Cells
GSCF	Granulocyte Colony-Stimulating Factor
iPSCs	Induced Pluripotent Stem Cells
MIP1A	Macrophage Inflammatory Protein 1 α
